# The ASCIZ-DYNLL1 Axis Is Essential for TLR4-Mediated Antibody Responses and NF-κB Pathway Activation

**DOI:** 10.1128/MCB.00251-21

**Published:** 2021-11-22

**Authors:** Rui Liu, Ashleigh King, David Tarlinton, Jörg Heierhorst

**Affiliations:** a St. Vincent’s Institute of Medical Research, Fitzroy, Victoria, Australia; b Department of Medicine at St. Vincent’s Hospital, University of Melbourne Medical School, Fitzroy, Victoria, Australia; c Department of Immunology and Pathology, Monash University, Melbourne, Victoria, Australia

**Keywords:** B-cell responses, BIM, DYNLL1, NF-κB, cell proliferation, immunization

## Abstract

Toll-like receptors (TLRs) and interleukin-1 (IL-1) receptors regulate immune and inflammatory responses by activating the NF-κB pathway. Here, we report that B-cell‐specific loss of dynein light chain 1 (DYNLL1, LC8) or its designated transcription factor ASCIZ (ATMIN) leads to severely reduced *in vivo* antibody responses to TLR4-dependent but not T-cell‐dependent antigens in mice. This defect was independent of DYNLL1’s established roles in modulating BIM-dependent apoptosis and 53BP1-dependent antibody class-switch recombination. In B cells and fibroblasts, the ASCIZ-DYNLL1 axis was required for TLR4-, IL-1‐, and CD40-mediated NF-κB pathway activation but dispensable for antigen receptor and tumor necrosis factor α (TNF-α) signaling. In contrast to previous reports that overexpressed DYNLL1 directly inhibits the phosphorylation and degradation of the NF-κB inhibitor IκBα, we found here that under physiological conditions, DYNLL1 is required for signal-specific activation of the NF-κB pathway upstream of IκBα. Our data identify DYNLL1 as a signal-specific regulator of the NF-κB pathway and indicate that it may act as a universal modulator of TLR4 (and IL-1) signaling with wide-ranging roles in inflammation and immunity.

## INTRODUCTION

Antibody production by B cells is a defining feature of adaptive and innate immune responses (1). Antigen-specific antibody production is initiated by B-cell receptor (BCR)‐mediated activation of B lymphocytes in concert with signal-specific coactivators, including the CD40 ligand (CD40L)/CD40-mediated interaction with T cells and ligation of Toll-like receptors (TLRs) by innate immune modulators (2). A range of different TLRs are expressed at different stages of B-cell development and contribute to B-cell maturation, immunoglobulin gene (*Ig*) gene diversification, antibody production, and cytokine production (3, 4).

Although the different TLRs recognize a range of distinctive exogenous and endogenous signals, they—and the interleukin-1 (IL-1) receptor—all converge on MyD88 and associated signaling adapters and thereby lead to the activation of the NF-κB and mitogen-activated protein kinase (MAPK) pathways (4, 5). Activation of the NF-κB pathway by IL-1R/TLRs involves a complex signaling cascade, including the protein kinases IRAK1/4, TAK1, IKK, and ubiquitin (Ub) ligases TRAF6 and LUBAC (5). Ultimately, phosphorylation of the inhibitor of NF-κB (IκBα) by IKKβ (6) leads to its degradation and release of trapped NF-κB/Rel transcription factors into the nucleus to alter the expression of hundreds of target genes (7). In humans, mutations in TLR signaling components are associated with increased susceptibility to bacterial infections, autoimmune disorders, and B-cell lymphomas (3, 4, 8). In mice, the predominant TLR of B cells is TLR4, which plays a critical role in the innate immune response against Gram-negative bacteria by recognizing the sepsis-inducing bacterial endotoxin lipopolysaccharide (LPS) (3, 9).

DYNLL1 (also known as LC8) is a homodimeric sequence-specific chaperone that promotes the ordered (typically homo-) oligomerization of more than a hundred protein targets (10, 11). We recently reported that conditional deletion of *Dynll1* or its designated transcription factor ASCIZ (also called ATMIN) during early B-cell development (via *Mb1-Cre*) leads to ∼10-fold reduced numbers of mature follicular B cells (B2 cells) and an >100-fold reduction of the initial pool of innate-like B-1a cells (12, 13). The B2 but not the B-1a cell developmental defects of ASCIZ- or DYNLL1-deficient mice could be suppressed by simultaneous deletion of the known DYNLL1 target BIM. In addition, DYNLL1 also contributes to the efficiency of immunoglobulin gene (*Ig*) class switch recombination (CSR) in mature B cells, where it modulates the oligomerization of the central DNA damage response protein 53BP1 (14).

DYNLL1 has previously been proposed to function as an inhibitor of the NF-κB pathway, based on findings that it can bind directly to the IκBα regulatory region *in vitro* (15) and block its phosphorylation by IKK and subsequent degradation required for NF-κB release in overexpression experiments (16). In contrast, we found here that DYNLL1 at physiological expression levels is essential for TLR4- and IL-1‐mediated activation of the NF-κB pathway in mouse B cells and fibroblasts. We show here that DYNLL1 acts upstream of IκBα by promoting the signal-specific activation of IKK and demonstrate that this is physiologically relevant for TLR4-mediated antibody responses *in vivo*.

## RESULTS

### DYNLL1 regulates B-cell activation in a signal-specific manner *in vitro*.

**FIG 1:**
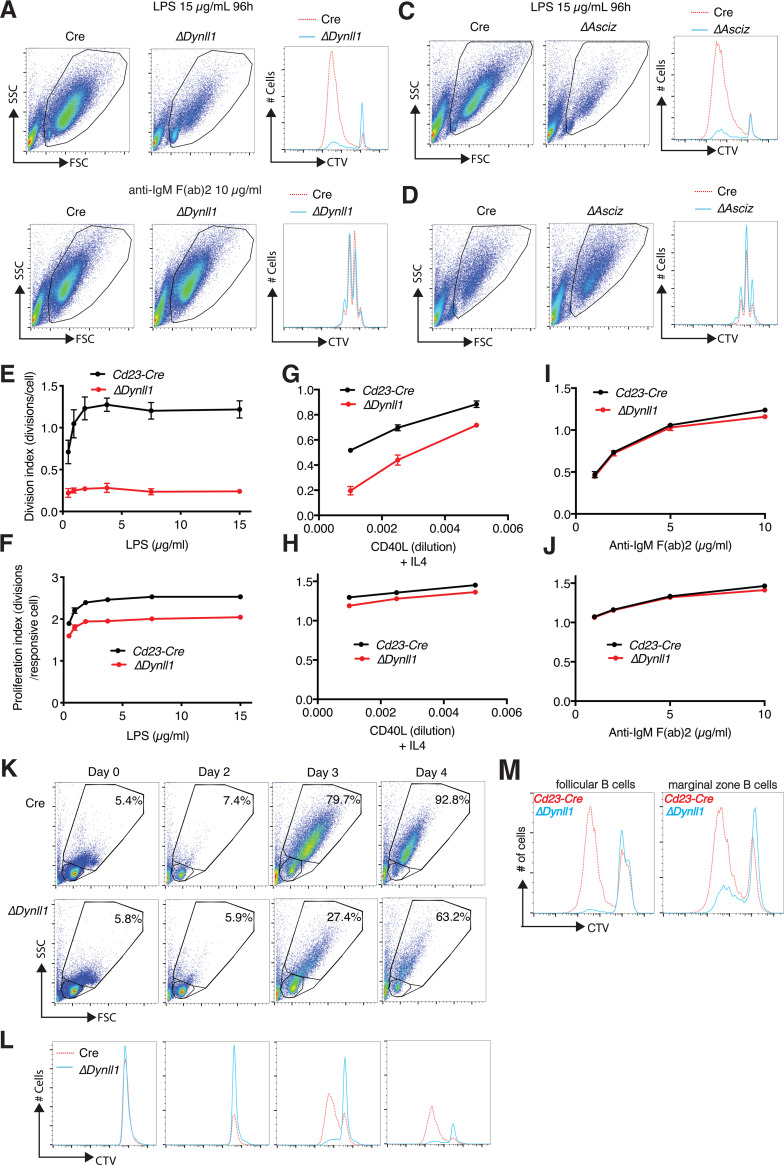
The ASCIZ-DYNLL1 axis is required for signal-specific B-cell activation. (A to D) Representative FACS plots and CellTrace Violet (CTV) dilution histograms of purified *Cd23-Cre* control, *Cd23-Cre Dynll1*-deleted, and *Cd23-Cre Asciz*-deleted splenic B cells after 4-day treatment with 15 μg/ml lipopolysaccharide (LPS) or 10 μg/ml anti-IgM F(ab)_2_ fragments as indicated. (E to J) Concentration-dependent responses of Cd23-Cre control and Cd23-Cre Dynll1-deleted B cells to LPS (E, F), CD40L (G, H), and BCR cross-linking (I, J) calculated from CTV FACS plots using FlowJo software. The cell division index (E, G, I) depicts the average number of divisions of all cells in the starting culture; the proliferation index (F, H, J) calculates the average number of divisions of cells that have divided at least once. Each data point represents the mean ± SEM of four independent biological replicates. (K) FACS plots of *Cd23-Cre* control and *Cd23-Cre Dynll1*-deleted splenic B cells treated for 0 to 4 days with 15 μg/ml LPS. The larger gate indicates the percentage of activated lymphoblasts, and the smaller gate represents lymphocytes. (L) CTV dilution profiles of the cultures (lymphocytes and lymphoblasts) shown above. (M) Comparison of CTV dilution profiles in FACS-purified follicular (CD23^hi^ and CD21^intermediate^) and marginal zone (CD23^low^ and CD21^hi^) B-cell fractions from *Cd23-Cre* control and *Cd23-Cre Dynll1*-deleted mice treated with 15 μg/ml LPS for 4 days. FSC, forward scatter; SSC, side scatter.

To investigate possible roles of DYNLL1 in mature B cells without confounding developmental defects, we used *Cd23-Cre* for its conditional deletion. This resulted in normal splenic B-cell numbers in *Cd23-Cre Dynll1*-deleted mice compared to those in *Cd23-Cre* controls (14). We then cultured purified B cells from these mice to assess their response to different activators *in vitro*. Stimulation with LPS led to the blasting of purified *Cd23-Cre* control B cells with a characteristic increase in cell size and extensive dilution of the proliferation-tracking dye CellTrace Violet (CTV) (Fig. 1A). In contrast, the emergence of larger lymphoblasts and CTV dilution was severely impaired in LPS-treated *Cd23-Cre Dynll1*-deleted B cells (Fig. 1A). Remarkably, in contrast to their severe activation defect in response to LPS, *Dynll1*-deleted B cells exhibited an entirely normal response to anti-IgM F(ab)_2_-mediated BCR activation (Fig. 1B). These results were independently confirmed with *Cd23-Cre Asciz*-deleted B cells (Fig. 1C and D), which also contain extremely low DYNLL1 levels (17).

Severe defects of *Dynll1*-deleted B cells in response to LPS (Fig. 1E) but normal responses to BCR cross-linking (Fig. 1I) were consistently observed over a wide range of ligand concentrations. A mixed effect was observed in response to CD40L, with severe activation defects at low doses but progressively improved proliferation with increasing CD40L concentrations (Fig. 1G).

The normal response to BCR stimulation and the concentration-dependent improvement of the response to CD40L suggested that the *in vitro* proliferation defects of DYNLL1-deficient B cells more likely reflect LPS/CD40L-specific signaling defects than intrinsic cell cycle or cell viability defects. This notion was supported by modeling of the CTV dilution profiles using the FlowJo cell proliferation software package; calculation of the average division number of all cells in the culture indicated severe reductions in the cell division index of LPS- or CD40L-treated DYNLL1-deficient cells (Fig. 1E and G). However, these defects were notably attenuated when only cells that had divided at least once were taken into consideration to calculate the proliferation index of these cultures (Fig. 1F and H). In addition, time course analyses indicated that increase in B-cell volume, which precedes cell proliferation of the larger lymphoblasts after LPS stimulation (9), was considerably reduced in *Cd23-Cre Dynll1*-deleted cells compared to in *Cd23-Cre* control cells (Fig. 1K and L). Finally, similar activation defects were observed in both the follicular and marginal zone B-cell fractions (Fig. 1M). Collectively, these results indicate that DYNLL1 is particularly important for the initial signal-specific response of B cells to LPS and CD40L, but once activated, DYNLL1-deficient B cells expand in a relatively normal manner.

### The ASCIZ-DYNLL1 axis is essential for TLR4-mediated antibody responses *in vivo*.

**FIG 2:**
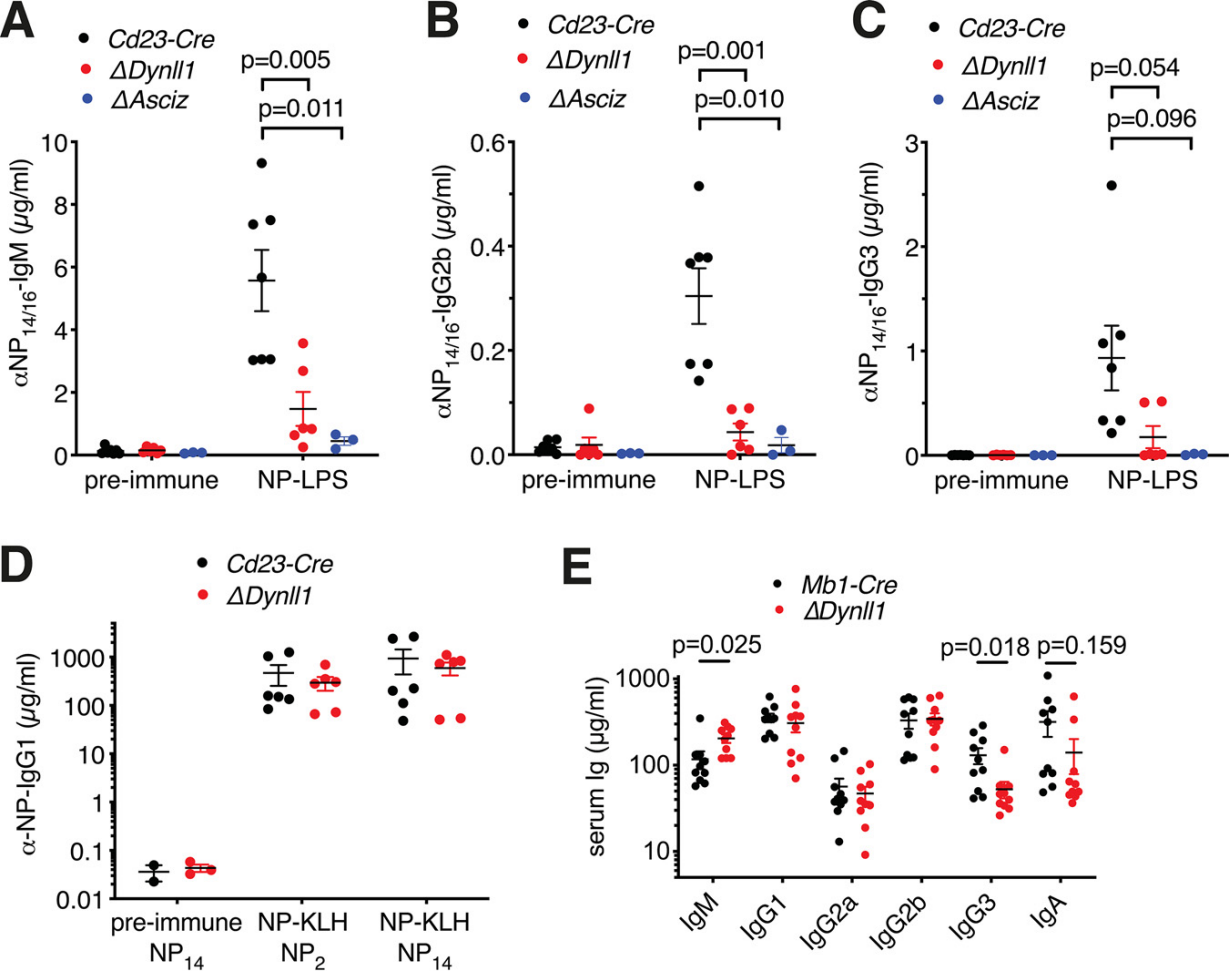
DYNLL1 is essential for TLR4-mediated T-cell-independent (TI-1) antibody responses but dispensable for T-cell-dependent antibody responses *in vivo*. (A to C) 4-Hydroxy-3-nitrophenyl-acetyl (NP)‐specific Ig titers before and 7 days after immunization with NP_10_-LPS (*n* = 3 to 7 mice per genotype). (D) Total (NP_14_) and high-affinity (NP_2_) antigen-specific IgG1 antibody titers (*n* = 6) 28 days after immunization with NP_21_-KLH. Preimmune sera depict separate mice. The results are from three separate immunization experiments. (E) Basal Ig titers (*n* = 10) of naive *Mb1-Cre* control and *Mb1-Cre Dynll1*-deleted mice at 8 weeks of age.

To determine whether the *in vitro* activation defects of ASCIZ- or DYNLL1-deficient B cells (Fig. 1) are physiologically relevant, we performed *in vivo* immunization experiments. In order to specifically determine the contribution of TLR4 signaling to the antibody response, we used LPS conjugated to the hapten 4-hydroxy-3-nitrophenyl-acetyl (NP) as a T-cell‐independent (TI-1) antigen to costimulate NP-specific B cells via their BCR and TLR4. In *Cd23-Cre* control mice, NP-LPS immunization led to >25-fold increased NP-specific IgM, IgG2b, and IgG3 antibody titers, but this response was severely attenuated in *Cd23-Cre Dynll1*- and *Cd23-Cre Asciz*-deleted mice (Fig. 2A through C). In contrast, in immunization experiments in which NP was conjugated to keyhole limpet hemocyanin as a T-cell‐dependent antigen, *Cd23-Cre Dynll1*-deleted mice had a largely normal antibody response (Fig. 2D), similar to what we have previously reported for *Cd23-Cre Asciz*-deleted mice (17). As the impaired TI-1 response includes unswitched NP-specific IgM titers (Fig. 2A), this defect is clearly independent of the recently reported role of DYNLL1 as an accessory factor in 53BP1-mediated antibody isotype switching (14). In this context, it should also be noted that conventional T-cell‐dependent antigen-specific IgG1 serum titers 4 weeks after *in vivo* immunization are largely maintained by selection and expansion of long-lived class-switched plasma cells (1), which would be expected to obscure the partial class-switch recombination defect of *Asciz*- or *Dynll1*-deleted B cells at much earlier time points *in vitro* (14).

We also measured basal Ig titers in naive mice. Because basal antibodies are believed to be produced by the innate-like B-1a cell lineage that lacks CD23 expression, we used *Mb1-Cre Dynll1*-deleted mice for this purpose. For most Ig isotypes, titers were indistinguishable between groups (Fig. 2E). However, *Mb1-Cre Dynll1*-deleted mice contained significantly higher IgM and significantly lower IgG3 titers than *Mb1-Cre* control mice (Fig. 2E), with a trend toward lower IgA titers.

Altogether, these data demonstrate that ASCIZ and DYNLL1 are specifically required for TLR4-mediated but largely dispensable for T-cell‐dependent antibody responses *in vivo*. The severe defect of the antibody response to NP-LPS is quantitatively comparable to the TLR4-dependent *in vitro* activation defects of *Dynll1*- or *Asciz*-deleted B cells. Conversely, considering that CD40L responses of DYNLL1-deficient B cells progressively improve in a concentration-dependent manner *in vitro*, the normal T-cell‐dependent antibody response is consistent with the notion that local CD40L concentrations are high during the direct interactions between T and B cells in *in vivo* (18). Finally, given that TLR4 signaling is particularly important for CSR from IgM to IgG3 (19), the specific reduction of basal IgG3 titers in naive *Mb1-Cre Dynll1*-deleted mice further supports an *in vivo* function of DYNLL1 in TLR4 signaling in B lymphoid cells.

### Differential rescue of DYNLL1-related *in vitro* activation but not *in vivo* immune defects by ablation of *Bim*.

An important facet of the TLR4 response during B-cell activation is the activation of antiapoptotic mechanisms, including induction of antiapoptotic genes and posttranslational inactivation of the proapoptotic protein BIM (20). Based on the established roles of DYNLL1 in directly restraining BIM activity *in vitro* and *in vivo* (12, 21, 22), we therefore sought to assess whether and how BIM contributes to B-cell activation and antibody response defects by generating *Cd23-Cre Dynll1 Bim*-double-deleted mice.

**FIG 3:**
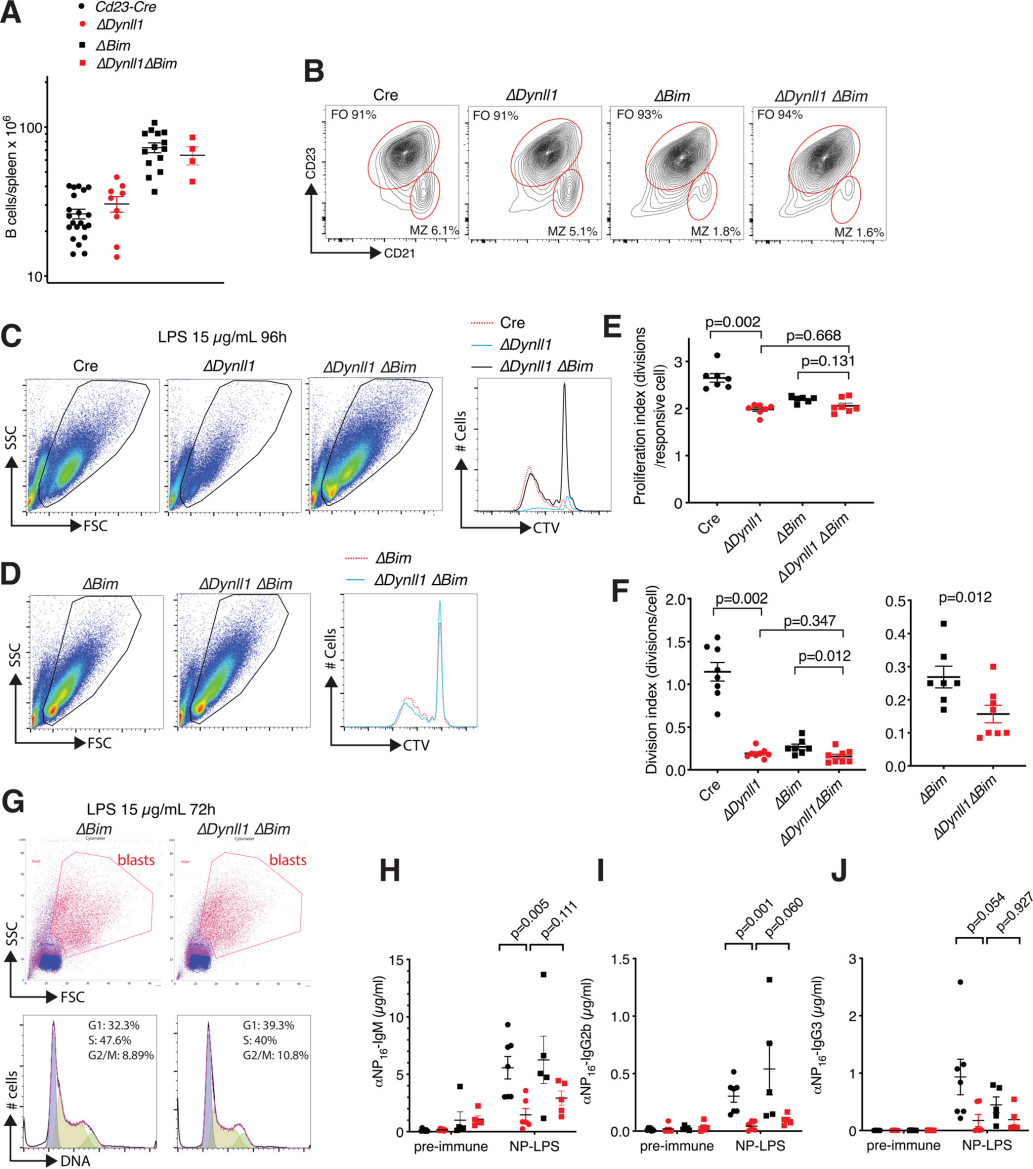
Differential effect of *Bim* deletion on *in vitro* activation and *in vivo* antibody responses of *Cd23-Cre Dynll1*-deleted B cells and mice. (A) Total splenic B-cell numbers (B220^+^ IgM^+^) of mice of the indicated genotypes (for *Cd23-Cre* versus *ΔDynll1*, *P* = 0.266; for *ΔBim* versus *ΔDynll1 ΔBim*, *P* = 0.001). (B) Representative FACS plots and quantification of follicular and marginal zone (MZ) B-cell fractions in spleens of mice with the indicated genotypes, indicating similar cellularity changes in *Cd23-Cre Bim*-deleted and *Cd23-Cre Dynll/Bim*-double-deleted mice and in control mice. (C, D) Representative FACS plots and CTV dilution curves of *Cd23-Cre* control, *Cd23-Cre Dynll1*-deleted, *Cd23-Cre Bim*-deleted, and *Cd23-Cre Dynll1/Bim*-double-deleted B cells treated with 15 μg/ml LPS for 96 h. (E, F) FlowJo-calculated proliferation and division indices of *Cd23-Cre* control, *Cd23-Cre Dynll1*-deleted, *Cd23-Cre Bim*-deleted, and *Cd23-Cre Dynll1/Bim*-double-deleted B cells treated with 15 μg/ml LPS for 96 h. The data for *Cd23-Cre Bim*-deleted and *Cd23-Cre Dynll1/Bim*-double-deleted cells are duplicated on two different *y* axis for clarity. (G) FACS plots of lymphoblast purifications and their cell cycle profiles after treatment with 15 μg/ml LPS for 72 h. (H to J) NP-LPS antibody responses in *Cd23-Cre Bim*-deleted and *Cd23-Cre Dynll1/Bim*-double-deleted mice compared to the results for *Cd23-Cre* control and *Cd23-Cre Dynll1*-deleted mice from Fig. 2A to C.

Similar to what we recently reported for *Cd23-Cre Bim*-deleted mice (23), *Cd23-Cre Dynll1 Bim*-double-deleted mice contained ∼2-fold expanded mature follicular B-cell numbers compared to *Cd23-Cre* control and *Cd23-Cre Dynll1*-deleted mice (Fig. 3A and B). Interestingly, compared to *Dynll1*-deleted B cells, concomitant loss of *Bim* led to considerably increased numbers of proliferating lymphoblasts in LPS-treated cultures (Fig. 3C and D). Comparison of the proliferation index of cells in these cultures that have divided at least once indicated no differences between *Bim*-deleted and *Dynll1/Bim*-double-deleted lymphoblasts (Fig. 3E). These results indicate that BIM-mediated apoptosis is a major factor in the reduced proliferation rates of *Dynll1*-deficient B cells in response to LPS. However, when considering all cells in the culture, it is important to note that loss of BIM led to a marked increase in the number of surviving nonresponsive small double-deleted lymphocytes, similar to the protective effect *Bim* deletion by itself has on the viability of activation-resistant B cells *in vitro* (23). Because of the increased survival of nonresponsive cells, *Bim* deletion did not affect the division index of *Dynll1*-deleted B cells calculated by the FlowJo program (Fig. 3F). However, compared to the >5-fold reduced division index of *Dynll1*-deleted cells relative to *Cd23-Cre* control cells, the division index of *Dynll1/Bim*-double-deleted cells was reduced by less than 2-fold relative to *Bim* single mutant cells (Fig. 3D and F). This relative improvement indicates that increased BIM-mediated apoptosis in the absence of DYNLL1 is an important factor in the *in vitro* activation defects of *Cd23 Dynll1*-deleted B cells, but the residual difference (*P* = 0.012) (Fig. 3F) between *Dynll1*-deleted and *Dynll1/Bim*-double-deleted cells indicates that BIM is not wholly responsible for this effect. In support of additional BIM-independent mechanisms, purified LPS-treated *Dynll1/Bim*-double-deleted lymphoblasts contained a higher proportion of G_1_-phase cells compared to *Bim* controls (Fig. 3G), consistent with decreased mitogenic responsiveness downstream of TLR4 activation.

To assess whether the partial rescue effect of *Bim* deletion on the activation of *Dynll1*-deficient B cells translates to improved antibody responses *in vivo*, we immunized *Cd23-Cre Bim*-deleted and *Cd23-Cre Dynll1 Bim*-double-deleted mice with NP-LPS. However, additional deletion of *Bim* only marginally—and to a much lower level than in *Cd23-Cre* and *Bim-deleted* control mice—improved the NP-specific IgM, IgG2b, and IgG3 antibody titers in *Dynll1*-deficient mice, and none of the small improvements were statistically significant (Fig. 3H to J). Thus, although increased BIM activity contributes to the *in vitro* activation defects of *Dynll1*-deficient B cells, their impaired antibody responses *in vivo* are largely mediated by other mechanisms.

### DYNLL1 is required for and upregulated in response to LPS-induced NF-κB pathway activation in B cells.

**FIG 4:**
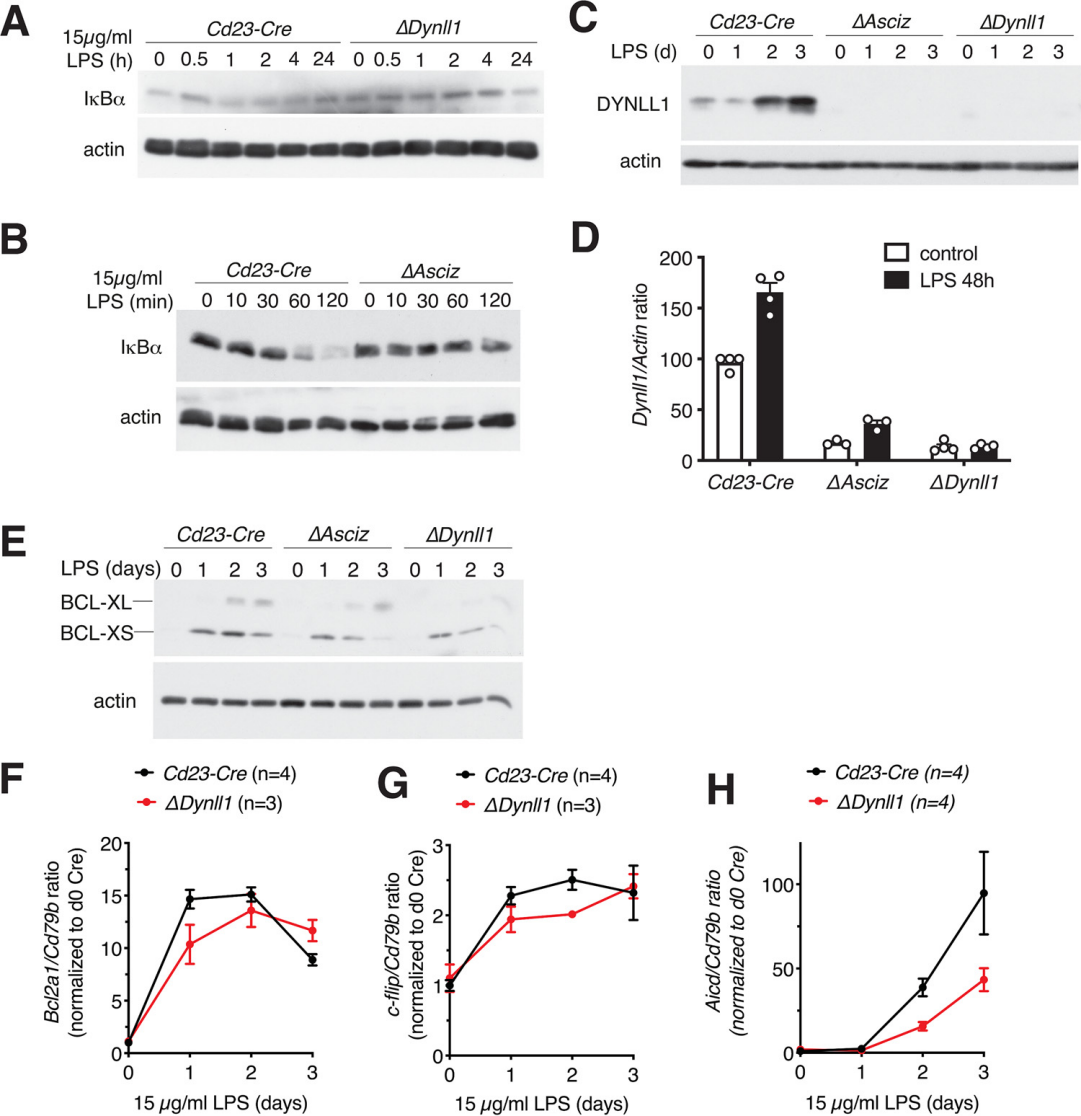
The ASCIZ-DYNLL1 axis is required for LPS-induced NF-κB pathway activation in B cells. (A to C) Western blot analysis of *Cd23-Cre* control, *Cd23-Cre Dynll1*-deleted, and *Cd23-Cre Asciz*-deleted splenic B cells treated for the indicated times with 15 μg/ml LPS. (D) Quantitative RT-PCR analysis of *Dynll1* mRNA expression, normalized to *Actin* mRNA, after treatment of *Cd23-Cre* control, *Cd23-Cre Dynll1*-deleted, and *Cd23-Cre Asciz*-deleted splenic B cell with 15 μg/ml LPS for 48 h (for *Cd23-Cre*, *n* = 4, *P* = 0.00004; for *ΔAsciz*, *n* = 3, *P* = 0.0071; for *ΔDynll1*, *n* = 4, *P* > 0.999). (E) Western blot analysis of the NF-κB target BCL-X-S/L in *Cd23-Cre* control, *Cd23-Cre Dynll1*-deleted, and *Cd23-Cre Asciz*-deleted splenic B cells treated for 0 to 3 days with 15 μg/ml LPS. (F to H) qPCR quantitation of NF-κB targets *Bcl2a1*, c-*flip*, and *Aicd* in *Cd23-Cre* control and *Cd23-Cre Dynll1*-deleted splenic B cells for 0 to 3 days with 15 μg/ml LPS.

Because LPS and TLR4 are prototypical activators of the canonical NF-κB pathway, our findings that DYNLL1 was essential for TLR4-mediated B-cell activation and antibody responses seemed contradictory to its previously proposed function as an inhibitor of IκBα degradation and NF-κB activation (16). To resolve this conundrum, we performed immunoblot analyses to assess IκBα protein levels in LPS-treated B cells. As expected, IκBα protein amounts were transiently reduced after 1- to 2-h LPS treatment in *Cd23-Cre* control B cells, but in contrast to the current model, IκBα levels remained largely stable in *Cd23-Cre Dynll1*-deleted B cells (Fig. 4A). These results were independently confirmed using *Cd23-Cre Asciz*-deleted B cells (Fig. 4B), which also lacked detectable DYNLL1 expression (Fig. 4C). Interestingly, we noticed that DYNLL1 itself was significantly upregulated—at both the protein and mRNA levels—during the LPS-induced lymphoblast differentiation (Fig. 4C and D).

We also monitored the response of selected NF-κB target genes (BCL-XL/S, *Bcl2a1*, c-*flip*, and *Aicd* genes) over a 3-day LPS treatment time course. While the targets exhibited different induction kinetics, in all cases their response was modestly delayed in *Cd23-Cre Dynll1*-deleted B cells compared to the response of *Cd23-Cre* controls (Fig. 4E to H). Collectively, these results indicate that in contrast to ectopic overexpression experiments in cell lines (16), under physiological conditions, including moderate upregulation during the LPS response, DYNLL1 may promote LPS-induced activation of the NF-κB pathway in primary B cells.

### DYNLL1 is required for signal-specific activation of the NF-κB pathway upstream of IκBα.

**FIG 5:**
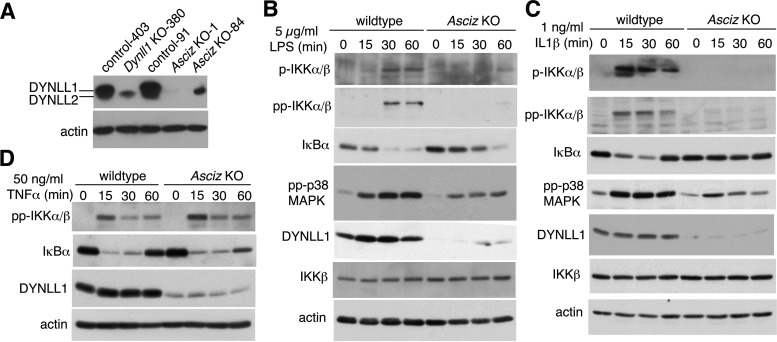
The ASCIZ-DYNLL1 axis is required for LPS- and IL-1–induced NF-κB pathway activation upstream of IκBα in MEFs. (A) Western blot analysis of DYNLL1 and DYNLL2 levels in isogenic control, *Dynll1^−/−^*, and *Asciz^−/−^* MEFs with a DYNLL1/2-cross-reactive antibody. Note that DYNLL2 has faster mobility than DYNLL1 and higher affinity for this antibody (25). MEF cell line numbers correspond to the originating embryo. (B to D) Western blots of isogenic wild-type control and DYNLL1-deficient *Asciz*^−/−^ MEFs (clone 1). The treatment conditions and antibodies used are indicated within the figure panels. IL1, interleukin-1; TNF-α, tumor necrosis factor α.

To expand our findings beyond B cells, we turned to fibroblasts. Because germ line loss of *Dynll1* leads to early embryonic lethality in mice, we used limb buds, which are highly enriched in fibroblasts, from mesenchyme-specific conditional *Prx1-Cre Dynll1*-deleted and *Prx1-Cre* control embryonic day 12.5 (E12.5) embryos to prepare murine embryonic fibroblast (MEF) cell lines (24). However, during the immortalization process, the closely related DYNLL2 protein (>95% amino acid identity to DYNLL1), which runs just below DYNLL1 on Western blots (25) and is normally expressed at >100-fold lower levels (24), was noticeably upregulated in *Dynll1^−/−^* MEFs. ASCIZ is an extremely specific transcription factor for the *Dynll1* gene with general congruence between *DYNLL1*-null and *ASCIZ*-null loss of function phenotypes from *Drosophila* to mammals (12, 14, 17, 24, 26–29). In order to avoid interference from the upregulated DYNLL2 in *Dynll1^−/−^* MEFs, we therefore chose an *Asciz*^−/−^ MEF clone that contained the lowest levels of DYNLL1 and DYNLL2 for these experiments (Fig. 5A) (note that only *Dynll1* and not *Dynll2* is directly regulated by ASCIZ [30]). Again, IκBα degradation in response to LPS treatment was notably attenuated in the *Asciz*^−/−^ cells compared to the response in isogenic wild-type control MEFs (Fig. 5B), demonstrating that the importance of the ASCIZ-DYNLL1 axis for TLR4 signaling extends beyond the B-cell lineage.

We also monitored activation of the IKK complex, which acts upstream of IκBα. Remarkably, while TAK1-mediated priming (p-IKK) and autoactivating diphosphorylation (pp-IKK) of IKK was evident after 30 to 60 min of LPS treatment in control cells, it was barely detectable in the DYNLL1-deficient *Asciz*^−/−^ MEFs (Fig. 5B). In addition to IKK, TAK1 also activates the p38 MAPK pathway in response to LPS treatment in a bifurcation from the NF-κB pathway (5, 6). Although activating p38 diphosphorylation (pp-p38) was still detectable in *Asciz*^−/−^ cells, it was notably attenuated compared to diphosphorylation in control MEFs (Fig. 5B). These results imply that DYNLL1 acts at the level of or upstream of TAK1 by either promoting TAK1 activation or its interaction with IKK and p38.

Comparable results were also obtained for treatment with IL-1β, whose receptor activates the NF-κB pathway similar to TLR4 via MyD88 as a shared signaling adaptor (5). Similar to the LPS response, IKK activation and IκBα degradation were severely impaired, and p38 activation was noticeably diminished in IL-1‐treated *Asciz*^−/−^ MEFs compared to p38 activation in control cells (Fig. 5C). Interestingly, in contrast to the impaired response to LPS and IL-1β, IKK activation and IκBα degradation was unaffected in *Asciz*^−/−^ MEFs in response to treatment with tumor necrosis factor-α (TNF-α) (Fig. 5D), the receptor for which does not interact with MyD88 and uses different signaling components to activate TAK1 and IKK (31). Taken together, these results indicate that, similar to its role in B-cell activation, the ASCIZ-DYNLL1 axis regulates the NF-κB pathway in MEFs in a signal-specific manner by promoting the activation of the TAK1-IKK cascade upstream of IκBα.

## DISCUSSION

**FIG 6:**
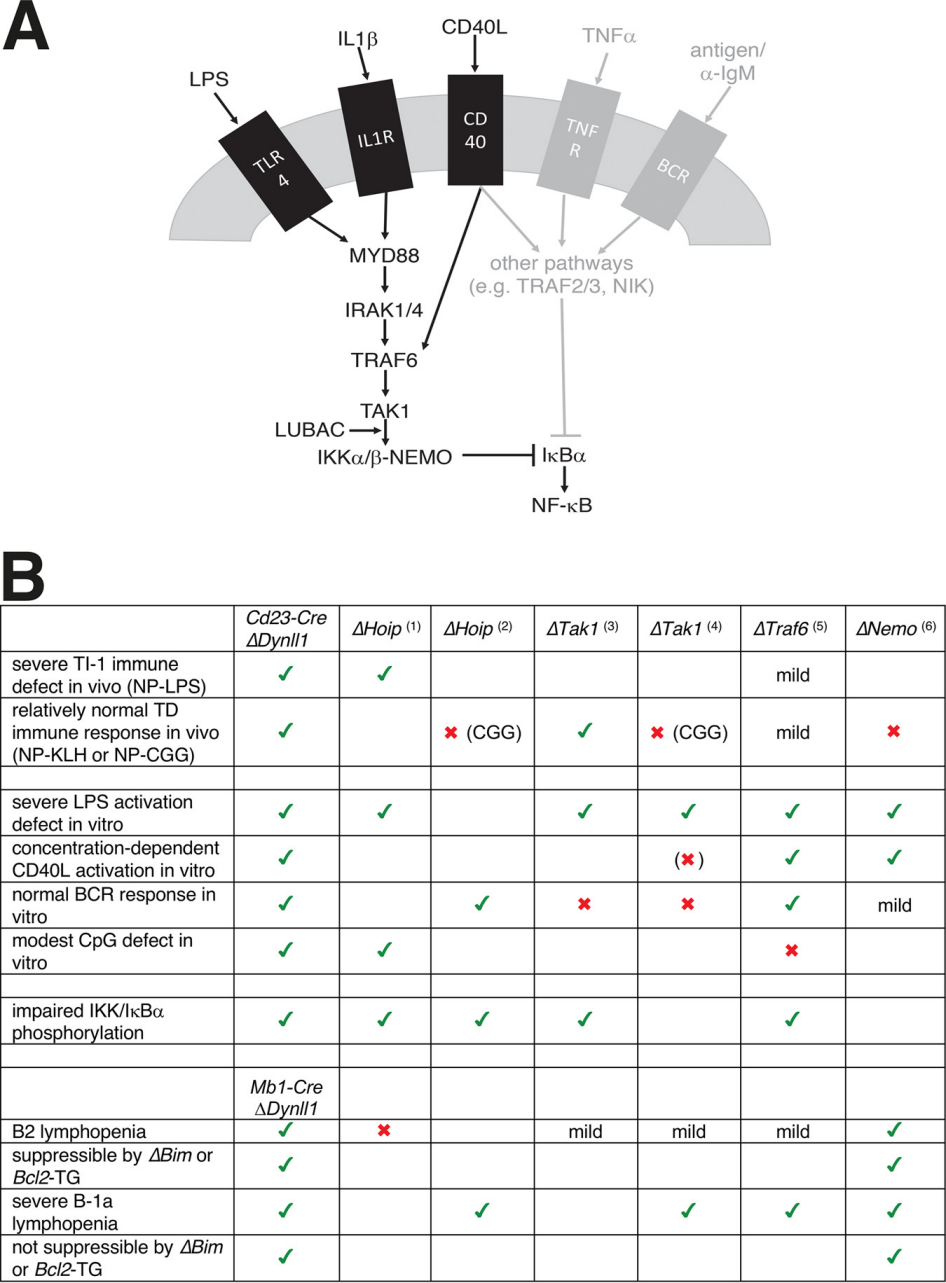
Role of the ASCIZ-DYNLL1 axis in NF-κB signaling. (A) Schematic diagram of ASCIZ/DYNLL1-dependent signaling in B cells and MEFs. Pathways that require DYNLL1 for IκBα degradation are shown in black, and DYNLL1-independent pathways are shown in gray. (B) Comparison of B-cell phenotypes of *Dynll1*-deficient mice to published *Traf6*, LUBAC (*Hoip1*), *Tak1*, and *Nemo* conditional KO mice. Green ticks indicate similar phenotypes; red crosses indicate different phenotypes. Phenotypes for other mice are compiled from *Mb1-Cre ΔHoip* (Sasaki et al. [37]), *Mb1-Cre ΔHoip* (Sasaki et al. [38]), *Cd19-Cre ΔTak1* (Schuman et al. [34]), *Mb1-Cre ΔTak1* (Shinohara et al. [35]), *Cd19-Cre ΔTraf6* (Kobayashi et al. [33]), and *Mb1-Cre* and *Cd21-Cre ΔNemo* (Derudder et al. [36]).

Collectively, our data from B cells and MEFs show that the ASCIZ-DYNLL1 axis is required for the signal-specific activation of the NF-κB pathway in response to LPS, IL-1, and low-dose CD40L but not to TNF-α and BCR ligation. A common and distinguishing feature of the affected pathways is that they signal at least in part through the Ub ligase TRAF6 (Fig. 6A). In contrast, the unaffected ligand TNF and the BCR pathway utilize the related Ub ligase TRAF2 or the TAK1-independent noncanonical NF-κB pathway, respectively (or both TRAF6 and TRAF2 in case of the partially impaired CD40L response), to promote IκBα phosphorylation and degradation (31). While the precise mechanism of DYNLL1’s action in this context remains to be determined, we speculate that it may contribute to the assembly of TRAF6‐TAK1‐IKK activation complexes. As DYNLL1 typically promotes the oligomerization of its targets by facilitating the alignment of their oligomerization domains, including often coiled-coil domains, into an ordered register (10, 11), it is interesting to note that coil-coiled mediated oligomerization has recently been shown to regulate TRAF6 catalytic processivity (32).

Consistent with the biochemical support for a role of DYNLL1 upstream of IκBα, the B-cell defects we have reported here are overall remarkably similar to the phenotypes of B lymphoid-specific conditional deletions of *Traf6* (33), *Tak1* (34, 35), the IKK subunit *Nemo* (36), and the LUBAC catalytic subunit *Hoip* (37, 38). These similarities (Fig. 6B) include severely impaired LPS-induced B-cell activation *in vitro* (*Traf6*, *Tak1*, *Nemo*, and *Hoip*); impaired IKK activation or IκBα degradation in response to LPS (*Traf6*, *Hoip*, and *Tak1*); concentration-dependent CD40L-induced activation defects (*Traf6*, *Tak1*, and *Nemo*); and impaired *in vivo* antibody responses to NP-LPS (*Traf6* and *Hoip*). The main differences between our findings and the other models relate to the *in vivo* response to T-cell‐dependent antigens, which were also largely normal in NP‐keyhole limpet hemocyanin (KLH)‐immunized *Tak1*-deleted mice but impaired in *Traf6*-deleted (albeit mildly), *Hoip*-deleted, or *Nemo*-deleted mice (as well as NP-CGG‐immunized *Tak1*-deleted mice), and *in vitro* responses to BCR ligation, which were impaired in the absence of *Tak1* or *Nemo* but also normal in *Hoip*- or *Traf6*-deleted B cells. It should be noted that the *Traf6*, *Tak1*, *Nemo*, and *Hoip* deletions were driven by *Mb1-Cre* or *Cd19*-Cre, which become active during early B lymphoid stages (39) and therefore also lead to developmental defects that may indirectly contribute to some of the differences compared to our postdevelopmental *Cd23-Cre*‐driven *Asciz* and *Dynll1* deletions (14, 17). Indeed, when considering the previously reported developmental defects of *Mb1-Cre Dynll1-* or *Asciz*-deleted mice (12, 13), the similarities between the mouse models extend even further. *Traf6-*, *Tak1-*, and *Nemo-*deleted mice also contain mildly to severely reduced B-2 cell numbers, which in the case of *Nemo* and *Asciz/Dynll1* deletion could be suppressed by transgenic BclII overexpression or *Bim* deletion, respectively; and *Traf6-*, *Tak1-*, *Nemo*-, and *Hoip*-deficient mice contain extremely reduced B-1a cell numbers, which similar to *Mb1-Cre Dynll1-* or *Asciz*-deleted mice cannot be rescued by the BclII transgene or *Bim* deletion.

Finally, the seemingly contradictory observations that DYNLL1 at physiological expression levels promotes but at excessively high overexpression levels inhibits NF-κB signaling (16) may highlight the intriguing dual activity of ASCIZ in the regulation of the *Dynll1* gene. While ASCIZ is essential for the activation of *Dynll1* expression, it also functions as a feedback sensor of DYNLL1 protein levels because DYNLL1 can bind directly to >10 different sites in the transcription activation domain of ASCIZ and thereby repress its activity (30, 40). It has remained an enigma why DYNLL1 requires such a designated bimodal transcription factor. The opposing effects of very low versus very high DYNLL1 protein levels on NF-κB signaling suggest that one important function of this feedback mechanism may be to ensure that the positive role of DYNLL1 in the upstream activation of the NF-κB pathway is not counteracted by an inadvertent inhibitory effect on the interaction of activated IKK with its target IκBα.

An important question for future work will be to determine the precise molecular mechanism of how DYNLL1 promotes the activation of IKK and which protein it binds to in this context. Finally, based on the similar findings for the important role of the ASCIZ-DYNLL1 axis in the regulation of TLR4-mediated NF-κB pathway activation in B cells and MEFs, we would predict that this role is universally conserved across different cell types. Given the importance of TLR4 signaling in inflammation and immunity, including sepsis, it will therefore be interesting to assess its functions in other cell types and disease processes.

## MATERIALS and METHODS

### Mice.

Animal experiments were performed according to the Australian Code for the Care and Use of Animals for Scientific Purposes, 8th Edition (2013), and approved by the St. Vincent’s Hospital Melbourne Animal Ethics Committee (approval 002/17). *Cd23-Cre* (41), *Mb1-Cre* (39), *Dynll1-floxed* (17), *Asciz-floxed* (13), and *Bim-floxed* (42) mice had been generated or backcrossed for at least 10 generations on a C57BL6 background and were housed in specific pathogen-free microisolators. *Cd23-Cre^Tg/+^ Dynll1^fl/+^* mice were intercrossed with *Dynll1^fl/+^* mice to generate *Cd23-Cre^Tg/+^ Dynll1^fl/fl^* deleted mice and isogenic *Cd23-Cre^Tg/+^ Dynll1^+/+^* controls. Similar heterozygous intercrosses were used to generate *Cd23-Cre Asciz*-deleted and *Mb1-Cre Dynll1-*deleted mice. For BIM experiments, *Cd23-Cre^Tg/+^ Dynll1^fl/+^ Bim^fl/fl^* mice were intercrossed with *Dynll1^fl/+^ Bim^fl/fl^* mice. Mice were used at ≥8 weeks of age for *in vitro* B-cell analyses and basal Ig titer assays and at 3 to 6 months of age for *in vivo* immunization experiments; they were not selected on gender.

### Immunization.

The mice were intraperitoneally (i.p.) injected with 10 μg NP_10_-LPS or with 100 μg NP_21_-KLH in alum. Serum was collected before, 1 week after (NP-LPS), and 4 weeks after (NP-KLH) immunization. NP-specific Ig titers were determined using Nunc Maxisorb plates coated with 50 μl per well of 5 μg/ml NP_2_‐bovine serum albumin (BSA) or NP_14_/NP_16_-BSA, and horseradish peroxidase (HRP)‐labeled goat anti-mouse IgM, IgG1, IgG2, or IgG3 antibodies (Southern Biotech, 5300-05). For quantification, reference wells were coated with goat anti-mouse Ig capture antibody and purified mouse IgM, IgG1, IgG2, or IgG3 (Southern Biotech, 5300-01). Enzyme-linked immunosorbent assay (ELISA) plates were developed using 2,2'-azinobis(3-ethylbenzothiazolinesulfonic acid) (ABTS) and read using a Polarstar at 410 nm.

### B-cell isolation and *in vitro* activation.

B cells were isolated from single-cell splenocyte suspensions using B-cell isolation kits (Miltenyi Biotec, 130090862) and magnetically activated cell sorting (MACS) separation LS columns (Miltenyi Biotec, 130042401) following the manufacturer’s instructions. Ten million isolated B cells were incubated with CellTrace Violet (CTV; Thermo Fisher Scientific, C34557) at 1:1,000 dilution at 37°C in the dark for 20 min and centrifuged at 300 × *g* for 10 min, and the cell pellet was washed once with 10 ml of MACS buffer. CTV-stained B cells were cultured in the B-cell culture media (RPMI 1640 [Sigma-Aldrich, R8758] supplemented with 5% (vol/vol) fetal bovine serum (FBS; Assay Matrix, ASFBS-F, batch A18C016], 50 μM β-mercaptoethanol, 100 U/ml penicillin G, and 100 μg/ml streptomycin sulfate) at a seeding density of 1 million cells/ml. The cells were stimulated with lipopolysaccharide (LPS; Invivogen, tlrl-3pelps), recombinant CD40L plus 1/100 conditioned mouse IL-4 supernatant (kind gift from Andreas Strasser, Walter and Eliza Hall Institute, Melbourne, Australia), or anti-IgM F(ab)_2_ fragment (eBioscience 16-5092-85) at the concentrations and treatment times indicated in the figure legends and panels.

### Flow cytometry.

The following reagents were used for cell staining: B220-FITC (Biolegend, 103206), CD19-APC eFluor780 (eBioscience, 47-0193-82), CD21-PE (Biolegend, 123410), CD23-biotin (BD, 553137), Brilliant Violet 605 Streptavidin (Biolegend, 405229), and propidium iodide (Sigma, P4864-10ML). Cells were applied to flow cytometry using a BD LSRFortessa with FACSDiva software (BD) and analyzed using FlowJo 10.3 software (Tree Star). Follicular and marginal zone B cells (Fig. 1M) and activated lymphoblasts (Fig. 3G) were isolated using a BD Influx cell sorter. For follicular and marginal zone B-cell proportions, splenic single cell suspensions were first gated on live B220^+^ CD19^+^ lymphocytes, followed by gating on CD21/35 and CD23 (follicular B cells: CD21/35^lo^ CD23^hi^; marginal zone B cells: CD21/35^hi^ CD23^lo^). Cell division and proliferation indices of CTV-stained cells were determined using the cell proliferation module, and cell cycle stages were determined using the cell cycle module of FlowJo 10.3 software (Tree Star).

### MEF experiments.

*Dynll1*^−/−^ MEFs and isogenic controls were prepared from E12.5 *Prx1-Cre Dynll1^fl/fl^* and *Prx1-Cre Dynll1^+/+^* embryonic limb buds (24) and immortalized randomly using the same standard 3T3 protocol as for the preparation of isogenic wild-type and *Asciz*^−/−^ MEFs from E12.5 embryos (43). MEFs were seeded at 1.5 × 10^5^ cells per well in 6-well plates in 3 ml Dulbecco’s modified Eagle’s medium (DMEM; Sigma-Aldrich, R8758) with 10% fetal calf serum (FCS) or at 10^6^ MEFs per 10-cm dish in 10 ml DMEM-FCS and grown for 2 days at 37°C with ambient O_2_ and 5% CO_2_. LPS, IL-1β (Propo Tech 200-01B-10UG), or TNF-α (In Vitro Technologies, RDS410TRNC010) was added with fresh medium at the doses and times indicated in the figure legends and panels.

### Western blots and quantitative PCR.

B cells were placed on ice at the indicated time points and collected by centrifugation at 375 × *g*, washed in ice-cold phosphate-buffered saline (PBS), and lysed in cold modified radioimmunoprecipitation assay (RIPA) buffer (150 mM NaCl, 20 mM Tris [pH 7.4] 1 mM EDTA, 1 mM EGTA, 10 mM NaF, 1% Triton X-100, 1% sodium deoxycholate, 0.1% SDS, 1 mM phenylmethylsulfonyl fluoride [PMSF], 1× protein inhibitor cocktail [Sigma]). MEFs were scraped directly into RIPA buffer on ice. Lysed cells were sonicated and centrifuged for 10 min at 4°C. Protein (∼25 μg per lane) was electrophoresed using Laemmli buffered SDS polyacrylamide gels and transferred to polyvinylidene difluoride (PVDF) membranes for detection using ECL (GE Healthcare) or Super-ECL reagents (Pierce) and X-ray film (Fuji). Films were scanned in *trans*-illumination mode using a dual lens Epson V700 photoscanner. The following antibodies were used: actin‐horseradish peroxidase (Santa Cruz Biotechnology, sc-1616, 1:10,000 dilution), BCL-X-L/S (BD Biosciences, 61021, 1:1,000), DYNLL1/2 (Abcam, ab51603, 1:5,000), IκBα (Cell Signaling Technology, 4814, 1:1,000), IKKβ (Cell Signaling Technology, 8943, 1:1,000), phospho-IKKα (S176)/IKKβ (S177) (Cell Signaling Technology, 2078, 1:1,000), di-phospho-IKKα/β (Ser176/180) (Cell Signaling Technology, 2697, 1:1,000), and phospho-p38 MAPK (Thr180/Tyr182) (Cell Signaling Technology, 4511, 1:1,000).

For quantitative PCR (qPCR) analyses, 3 × 10^6^ MACS-purified B cells were cultured with LPS (15 μg/ml) for 0 to 3 days. After treatment, the cells were washed once with ice-cold PBS, and cell pellets were snap-frozen on dry ice and stored at −80°C before RNA extraction. Total RNA was extracted using an ISOLATE II RNA micro kit (Bioline BIO-52075) following the manufacturer’s protocol. 300 ng purified RNA was subjected to reverse transcription with oligo(dT) and random primers using the SuperScript III first-strand synthesis system (Invitrogen, 18080-051). qPCR reagents were mixed as follows: 4 μl cDNA (6 ng), 0.5 μl primer mix (300 nM each), 5 μl SYBR Select Master mix (Applied Biosystems, 4472908), and 0.5 μl RNase/DNase free water. qPCR conditions were set as follows: 1 cycle (10 min at 95°C), 45 cycles (15 s at 95°C), and 60 s at 60°C. After each PCR run, a disassociation curve program was added to validate the primer specificity. The standard curve method of comparative quantification was employed to quantify the results (44). The results were normalized to *Cd79b* as an internal control and expressed as percentages of maximum expression. The following primers were used (37, 45): *Cd79b*_forward (5′-TGTTGGAATCTGCAAATGGA-3′), *Cd79b*_reverse (5′-TAGGCTTTGGGTGATCCTTG-3′); *Aicd*_forward (5′-AGAAAGTCACGCTGGAGACC-3′), *Aicd*_reverse (5′-CTCCTCTTCACCACGTAGCA-3′), *Bcl2a1a*_forward (5′-TCCACAAGAGCAGATTGCCCTG3′), *Bcl2a1a*_reverse (5′-GCCAGCCAGATTTGGGTTCAAAC-3′), c-*flip*_forward (5′-GCTCTACAGAGTGAGGCGGTTT-3′), and c-*flip*_reverse (5′-CACCAATCTCCATCAGCAGGAC-3′).

### Statistical analysis.

Data from independent experimental replicates were analyzed using GraphPad Prism software. The numbers of independent samples are indicated in the figures. Error bars indicate the mean ± the standard error of the mean (SEM). The *P* values were calculated using the two-tailed unpaired Student’s *t* test.
